# Integrative systematics and evolutionary history of *Berylmys bowersi* (Mammalia, Rodentia, Muridae)

**DOI:** 10.1002/ece3.10234

**Published:** 2023-07-04

**Authors:** Yifan Xu, Jiangxiao Hu, Zifan Shi, Wenwen Chen, Jiajun Zhou, Baowei Zhang, Fan Yong, Laxman Khanal, Xuelong Jiang, Zhongzheng Chen

**Affiliations:** ^1^ Collaborative Innovation Center of Recovery and Reconstruction of Degraded Ecosystem in Wanjiang Basin Co‐founded by Anhui Province and Ministry of Education, School of Ecology and Environment Anhui Normal University Wuhu China; ^2^ State Key Laboratory of Genetic Resources and Evolution & Yunnan Key Laboratory of Biodiversity and Ecological Security of Gaoligong Mountain, Kunming Institute of Zoology Chinese Academy of Sciences Kunming China; ^3^ School of Resources and Environmental Engineering Anhui University Hefei China; ^4^ Zhejiang Forest Resources Monitoring Center Hangzhou China; ^5^ School of Life Sciences Anhui University Hefei China; ^6^ Nanjing Institute of Environmental Sciences Ministry of Ecology and Environment Nanjing China; ^7^ Central Department of Zoology, Institute of Science and Technology Tribhuvan University Kathmandu Nepal

**Keywords:** *Berylmys latouchei*, eastern China, Murinae, Wuyi Mountains

## Abstract

The Bower's Berylmys (*Berylmys bowersi*) is one of the largest rodent species with a wide distribution range in southern China and the Indochinese Peninsula. The taxonomy and evolutionary history of the *B. bowersi* is still controversial and confusing. In this study, we used two mitochondrial (*Cyt b* and *COI*) and three nuclear (*GHR*, *IRBP*, and *RAG1*) genes to estimate the phylogeny, divergence times, and biogeographic history of *B. bowersi*. We also explored morphological variations among the specimens collected across China. Our phylogenetic analyses indicated that the traditional *B. bowersi* contains at least two species: *B. bowersi* and *B. latouchei*. *Berylmys latouchei* was considered a junior synonym of *B. bowersi* distributed in eastern China, which is confirmed to be distinguishable at specific level because of its larger size, relatively larger and whiter hind feet, and several cranial traits. The estimated split of *B. bowersi* and *B. latouchei* was at the early Pleistocene (ca. 2.00 Mya), which might be the outcome of the combined effects of climate change in the early Pleistocene and isolation by the Minjiang River. Our results highlight the Wuyi Mountains in northern Fujian, China, as a glacial refugia during the Pleistocene and call for more intensive surveys and systematic revisions of small mammals in eastern China.

## Abstract 2

The Bowers Berylmys (*Berylmys bowersi*) is one of the largest rodent species with a wide distribution range in southern China and the Indochinese Peninsula. Our analyses indicated that the traditional *B. bowersi* is a species complex contains at least two species: *B. bowersi* and *B. latouchei*. Also, our results highlight the Wuyi Mountains as the glacial refugia in the Pleistocene and call for more intensive surveys and systematic revisions of small mammals in the eastern China.

## 1 INTRODUCTION

White‐toothed rats (genus *Berylmys* Ellerman, 1947) are large rodents in the family Muridae that are widely distributed across Indochinese Peninsula, north‐east India, and southern China (Denys et al., 2017). They are patchily distributed in various habitats, including primary and secondary forests, and plantations. *Berylmys* was originally described as a subgenus of *Rattus* by Ellerman (1947) with *R. manipulus* (Thomas, 1916) and *R. berdmorei* (Blyth, 1851) in it. Yet, Ellerman (1961) assigned *R. bowersi* (Anderson, 1878) to the subgenus *Stenomys*, and regarded *mackenziei* (Thomas, 1916) as a subspecies of *R. bowersi*. Musser and Newcomb (1983) reviewed the taxonomy of *Berylmys* and promoted it to full generic status, containing four species: *B. manipulus*, *B. berdmorei*, *B. bowersi*, and *B. mackenziei*; a treatment that was widely accepted subsequently (Agrawal, 2000; Denys et al., 2017; Musser & Carleton, 2005).

The Bower's Berylmys (*Berylmys bowersi*; Musser & Carleton, 2005) is the largest and most widely distributed species of the genus and has been recorded in southern China, Vietnam, Myanmar, northern Laos, north‐eastern Thailand, India, and the Malay Peninsula (Denys et al., 2017). It is reported to live in burrows and is a habitat generalist inhabiting temperate and subtropical montane forests, disturbed forests, and abandoned jhum, cultivated fields, and moist deciduous and evergreen forests (Musser & Newcomb, 1983). It is a destructive agricultural pest in cereal and a reservoir or vector of numerous zoonotic diseases including scrub typhus, leptospirosis, and cryptosporidiosis (Boey et al., 2019; Chaisiri et al., 2017; Chen et al., 2019). A study on the evolutionary history and dispersal of *B. bowersi* is therefore important for public health and biodiversity conservation. Taxonomy of *B. bowersi* is still controversial due to limited research. It was described by Anderson (1879) based on specimens collected from the Kakyhen Hills of Husa (= Hotha), Dehong, Yunnan Province in China. Six other scientific names have been applied to samples of the species: including *Mus latouchei* Thomas, 1897; *Mus ferreocanus* Miller, 1900; *Rattus kennethi* Kloss, 1919; *Rattus bowersii lactiiventer* Kloss, 1919; *Rattus wellsi* Thomas, 1921; and *Rattus bowersii totipes* Tien, 1966; but all of them are currently treated as junior synonyms of *B. bowersi* (Denys et al., 2017; Wei et al., 2021).

Recently, we collected a specimen of *B. bowersi* in Qingliang Mountains, Anhui, eastern China. Our preliminary molecular analysis revealed that this specimen has a relatively high genetic distance with *B. bowersi* from Yunnan Province in southwestern China (Pei et al., 2022). Such a high genetic distance suggested underestimated diversity in the genus *Berylmys*. In the present study, we collected *Berylmys* specimens across China and sequenced two mitochondrial and three nuclear genes. By analyzing morphometric data and integrating newly generated sequences with the GenBank data, we assessed the taxonomy, phylogeny, and evolutionary history of *B. bowersi*.

## 2 MATERIALS AND METHODS

### 2.1 Sampling

**FIGURE 1:**
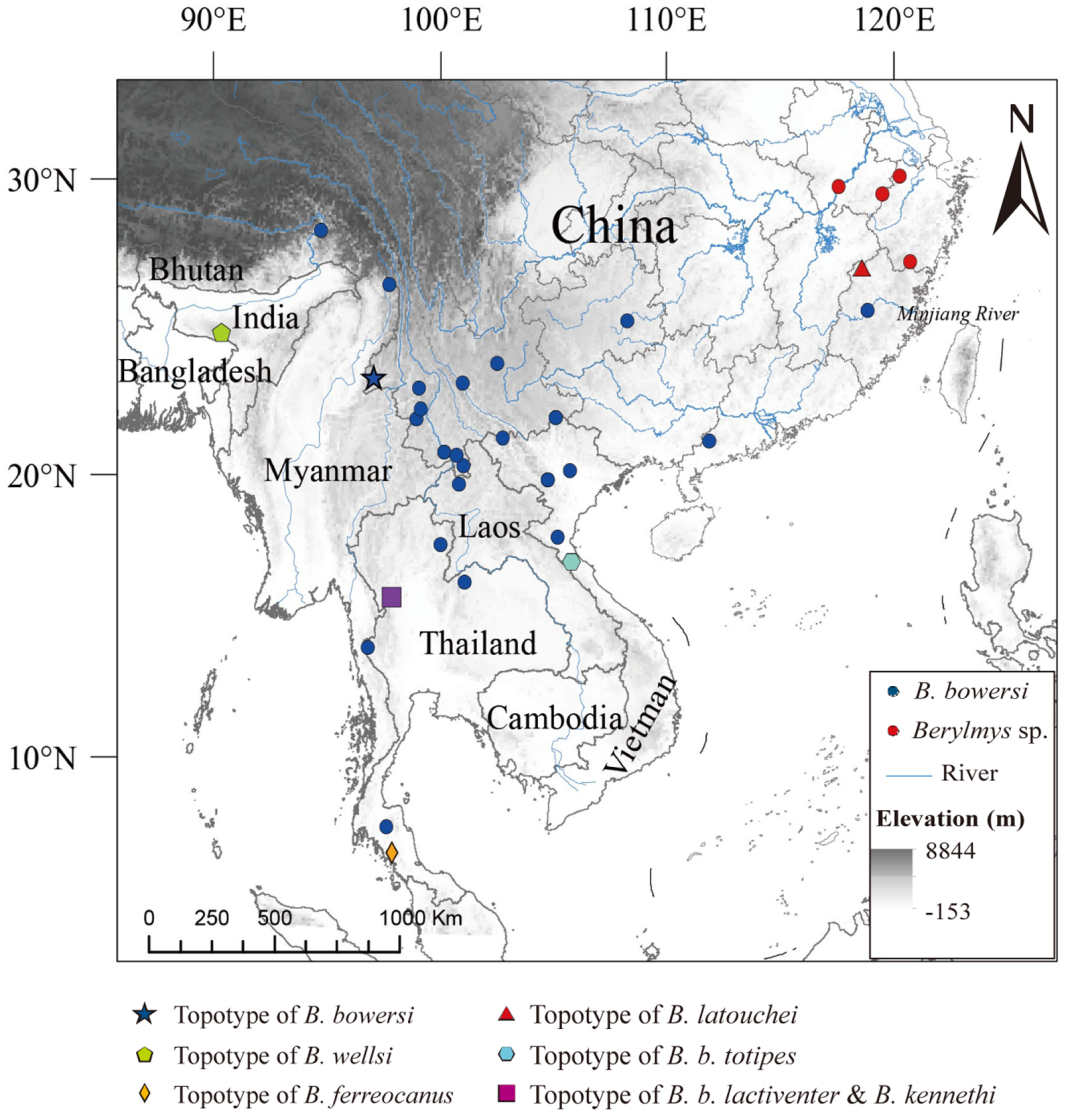
Sampling localities of specimens used in the phylogenetic analysis.

Animals were handled complying with the animal care and use guidelines of the American Society of Mammologists (Sikes et al., 2016), and following the guidelines and regulations approved by the internal review board of Anhui Normal University (with no special approval number), and with the permissions of local government authorities. Specimens and tissues were deposited at Anhui Normal University, Anhui, China, and Kunming Institute of Zoology (KIZ), Chinese Academy of Sciences, Kunming, China. Specimens were identified based on their morphology and distributions following Thomas (1897), Musser and Newcomb (1983), and Smith & Xie (2009). We obtained 41 *Berylmys* specimens across China (Figure 1). The specimen information and location data are listed in Table 1. Because *B. bowersi* from the eastern and the western China are genetically distinguishable from each other (Pei et al., 2022), we recognized only the western populations (including topotypic specimens of *B. bowersi*, *n* = 34) as *B. bowersi*, and assigned the eastern populations (*n* = 7) from the Anhui, Zhejiang, eastern China to an indeterminate species (*Berylmys* sp.).

**TABLE 1:** Information of samples used for phylogeny analysis.

Number	Species	Locality	*Cyt b*	*COI*	*IRBP*	*RAG1*	*GHR*
QLF1911356	*Berylmys* sp.	Xuancheng, Anhui, China	OQ160978	OQ168375	OQ161038	OQ161023	OQ160994
AD057	*Berylmys* sp.	Anqing, Anhui, China	OQ160990	OQ168387	OQ161049	OQ161034	OQ161004
AD113	*Berylmys* sp.	Anqing, Anhui, China	OQ160989	OQ168386	OQ161048	OQ161033	OQ161003
AD232	*Berylmys* sp.	Anqing, Anhui, China	OQ160988	OQ168385	OQ161047	OQ161032	OQ161002
AJ002	*Berylmys* sp.	Huzhou, Zhejiang, China	OQ160987	OQ168384	OQ161046	OQ161031	
AJ003	*Berylmys* sp.	Huzhou, Zhejiang, China	OQ160986	OQ168383			
HZ001	*Berylmys* sp.	Lishui, Zhejiang, China	OQ160980	OQ168377	OQ161040	OQ161025	OQ160996
AL1311278	*B. bowersi*	Yuxi, Yunnan, China	OQ160985	OQ168382	OQ161045	OQ161030	OQ161001
AL1311331	*B. bowersi*	Yuxi, Yunnan, China	OQ160984	OQ168381	OQ161044	OQ161029	OQ161000
AL1311911	*B. bowersi*	Yuxi, Yunnan, China	OQ160983	OQ168380	OQ161043	OQ161028	OQ160999
BN1409087	*B. bowersi*	Xishuangbanna, Yunnan, China	OQ160982	OQ168379	OQ161042	OQ161027	OQ160998
MT201705274	*B. bowersi*	Motuo, Xizang, China	OQ160981		OQ161041	OQ161026	OQ160997
LC201705207	*B. bowersi*	Puer, Yunnan, China	OQ160979	OQ168376	OQ161039	OQ161024	OQ160995
WS201703100	*B. bowersi*	Wenshan, Yunnan, China	OQ160976	OQ168373	OQ161036	OQ161021	OQ160992
WS201703143	*B. bowersi*	Wenshan, Yunnan, China	OQ160977	OQ168374	OQ161037	OQ161022	OQ160993
201403497	*B. bowersi*	Maoming, Guangdong, China	OQ160975	OQ168372	OQ161035	OQ161020	OQ160991
FJ001	*B. bowersi*	Sanming, Fujian, China		OQ168378			
MB9	*B. bowersi*	Son La, Vietnam	JN105099	JN105107	JN105091		
MB8	*B. bowersi*	Son La, Vietnam	JN105098	JN105106	JN105090		
MB3	*B. bowersi*	Son La, Vietnam	JN105097	JN105105	JN105089		
MB10	*B. bowersi*	Son La, Vietnam		JN105108	JN105092		
MB2	*B. bowersi*	Son La, Vietnam	JN105096	JN105104			
MVZ186482	*B. bowersi*	Vinh Phu, Vietnam	KC878024	KC878201		DQ019056	
L0151	*B. bowersi*	Luang Prabang, Laos	HM217477		HM217714		
R5410	*B. bowersi*	Nan, Thailand	HM217471		HM217707		
R4400	*B. bowersi*	Loei, Thailand	HM217453		HM217690		
R3425	*B. bowersi*	Loei, Thailand	HM217415		HM217653		
R3415	*B. bowersi*	Loei, Thailand	HM217413		HM217651		
R3268	*B. bowersi*	Kanchanaburi, Thailand	HM217412		HM217650		
HCOBBO01	*B. bowersi*	Nakhon Si Thammarat, Thailand			KC010296		
KIZ PM26	*B. bowersi*	Nghe An, Vietnam	JX573336				
KIZ PM17	*B. bowersi*	Nghe An, Vietnam	JX573335				
47Bbo_28	*B. bowersi*	Loei, Thailand	JN675478				
Outgroup	*Mus pahari*		AB096839	KY605337	AJ698893	AB125844	KC953280
	*Rattus exulans*		JX534033	KC617850	AY326105		DQ019074
	*Niviventer cremoriventer*		KJ607284	JF459850	KC953417		DQ019067
	*Apodemus agrarius*		AB303226	MW585697	AB096845	AB303232	DQ019054
	*Micromys minutus*		OQ576169	OQ576282	OQ576232	OQ576201	OQ561709
	*Leopoldamys edwardsi*		MK123054	MK123007	MK123116	MK123211	MK123232
	*B. berdmorei*		HM217432	KC010295	HM217668		
	*B. manipulus*		AB973112				

### 2.2 DNA sequencing, phylogeny, and molecular dating

The total genomic DNA of 17 specimens including 7 *Berylmys* sp. and 10 *B. bowersi* was extracted using a DNA extraction kit (Tiangen DNeasy Blood and Tissue Kit). Two mitochondrial genes (cytochrome *b* [*Cyt b*] and cytochrome *c* oxidase subunit I [*COI*]) and three nuclear genes (interphotoreceptor retinoid‐binding protein [*IRBP*], recombination activating 1 [*RAG1*], and growth hormone receptor [*GHR*]) were amplified using the primer pairs provided in Table 2. The PCR products were purified and sequenced in both directions using the BigDye Terminator Cycle Kit v.3.1 (Invitrogen) on an ABI 3730xl sequencer (Applied Biosystems). Sequences of all genes were edited using SeqMan 7.1. Each gene was aligned using MEGA 11 (Tamura et al., 2021) and then checked manually. All new sequences were deposited in GenBank (Accession numbers OQ160975–OQ161004, OQ161020–OQ161049, OQ168972–OQ168387, Table 1). In addition, 32 corresponding sequences of 16 specimens were also downloaded from GenBank (Table 1). Homologous sequences of *Mus pahari*, *Rattus exulans*, *Niviventer cremoriventer*, *Apodemus agrarius*, *Micromys minutus*, *Leopoldamys edwardsi*, *Berylmys berdmorei*, and *Berylmys manipulus* were downloaded as outgroup taxa (Table 1). Maximum likelihood (ML) and Bayesian inference (BI) analyses were performed in PhyloSuite (Zhang et al., 2020) to construct the phylogenetic relationships. Three concatenated datasets were used for the phylogenetic analyses: (1) mitochondrial genes (mtDNA, 1830 bp); (2) nuclear genes (nDNA, 3120 bp), and (3) mitochondrial‐nuclear genes (mtDNA + nDNA, 4950 bp). The best‐fit partitioning schemes were estimated using PartitionFinder v.2.0 (Lanfear et al., 2012). The SH‐aLRT support values ≥80, ultrafast bootstrap values (UFBoot) ≥ 95, and posterior probabilities (PP) ≥ 0.95 were considered as strong supports (Huelsenbeck & Rannala, 2004; Minh et al., 2019).

**TABLE 2:** Primers and PCR conditions for amplification and sequencing used in the genetic analyses.

Genes	Primer name	Primer sequences (5′ to 3′)	Annealing temperature (°C)	Reference
*Cytb*	*L14724_hk3*	GGACTTATGACATGAAAAATCATCGTTG	55	He et al. (2010)
	*H15915_hk3*	GATTCCCCATTTCTGGTTTACAAGAC		
*COI*	*BatL5310*	CCTACTCRGCCATTTTACCTATG	53	Robins et al. (2007)
	*R6036R*	ACTTCTGGGTGTCCAAAGAATCA		
*IRBP*	*IRBP A*	ATGGCCAAGGTCCTCTTGGATAACTACTGCTT	50	Cheng et al. (2017)
	*IRBP B*	CGCAGGTCCATGATGAGGTGCTCCGTGTCCTG		
*RAG1*	*RAG1‐F1705*	GCTTTGATGGACATGGAAGAAGACAT	55	Teeling et al. (2000)
	*RAG1‐R2915*	GAGCCATCCCTCTCAATAATTTCAGG		
*GHR*	*GHR5*	GGCRTTCATGAYAACTACAAACCTGACYTC	60	Galewski et al. (2006)
	*GHR6*	GAGGAGAGGAACCTTCTTTTTWTCAGGC		

Divergence times were estimated using the concatenated mitochondrial‐nuclear genes (mtDNA + nDNA) in BEAST v2.6.6 (Bouckaert et al., 2014). Because the missed genes will lead to inaccurate estimates of branch lengths and divergence times, we only used those samples with more than three sequences. Data blocks were defined based on genes and codon positions and evolutionary models or partition schemes were estimated based on the Bayesian Information Criterion (BIC). Three fossil calibration priors were used to provide for estimates of divergence times: (1) the most recent common ancestor (MRCA) among the “core” Murinae (12.5 Mya; 95% HPD: 11.31–13.99 Mya), with a lognormal distribution prior (mean: 12.5, standard deviation: 0.07, offset: 0), so the median age was at 12.5 Mya and the 95% CI was 11.1–14.0 Mya; (2) the major split occurred in the Indomalaya between Rattini and the remaining tribes of Murinae (11.2 Mya; 95% HPD: 9.91–12.67 Mya), also with a lognormal distribution prior (mean: 11.2, standard deviation: 0.08, offset: 0), so the median age was 11.2 Mya and the 95% CI was 9.8–12.7 Mya (Aghova et al., 2018); and (3) the oldest fossil of *B. bowersi*, which dated at 1.8 Mya, with an exponential distribution prior (offset = 1.8, *M* = 0.6 [1.8 × 0.333]), so the median age was 2.22 Mya and the 95% CI was 1.8–3.6 Mya (Zheng, 1993). Each Bayesian analysis was composed of a random starting tree, a lognormal relaxed molecular clock model, and a birth‐death tree prior. Each analysis was run for 100 million generations, sampling every 10,000 generations. The first 10% of the samples were discarded as burn‐in. Convergence was assessed using Tracer v1.7 (Rambaut et al., 2018).

### 2.3 Species delimitation

The uncorrected *p*‐distance of *Cyt b* gene was calculated in MEGA 11 to make pairwise comparisons of genetic differentiation within and between different phylogenetic lineages. BPP v3.1 was used for molecular species delimitation, employing both A10 (Yang & Rannala, 2010) and A11 models (Yang & Rannala, 2014). Based on the phylogenetic trees, we assigned *B. bowersi* and *Berylmys* sp. as two candidate species and used the best BI tree as the guide in the Bayesian phylogenetics and phylogeography (BPP) analysis. To avoid poor mixing during BPP analyses, we followed the suggestions of Yang (2015) and used the combinations of algorithm 0, algorithm 1, and different priors for models A10 and A11, respectively. “Heredity = 1” allowing θ (referred to as “thetaprior”) to vary among loci, or “Locus rate = 1” specifying the random‐rates model of Burgess and Yang (Burgess & Yang, 2008), were also used but not at the same time. In total, 24 individual BPP analyses were performed (Table 4), and each was repeated two times.

### 2.4 Morphological measurements and statistical analyses

External measurements including head and body length (HB), tail length (TL), hindfoot length without claws (HF), and length of ear from intertragal notch to crown (EL) were taken in the field with a ruler to the nearest 0.1 mm. The body weight (W) of each specimen was weighed to the nearest 0.01 g using an electronic scale. Twenty‐three craniodental measurements were taken to the nearest 0.01 mm for 32 skulls of adult individuals, using a digital caliper graduated to 0.01 mm following Pan et al. (2007). The craniodental measurements included greatest length of skull (GLS), palatal length (PL), postpalatal length (PPL), length of palatal bridge (LPB), length of incisive foramina (LIF), zygomatic width (ZMW), braincase width (BCB), mastoid width (MTW), interorbital breadth (IOB), maximum width across the upper second molars (M^2^–M^2^), the least internal breadth between first molars (BM^1^), rostrum width (RSW), length of upper tooth row (LUTR), length of upper molars (LUM), nasal length (NSL), braincase height (BCH), length of auditory bulla (LAB), distance between auditory bulla (DAB), greatest breadth of the foramen magnum (GBFM), mandibular length (ML), length of lower tooth row (LLTR), length of lower molars (LLM) and height of coronoid process (HCP). Thomas (1897), and Musser and Newcomb (1983) were followed for the terminology of morphological descriptions. All the craniodental measurements were taken by Zhongzheng Chen.

To evaluate the morphological variation between *B. bowersi* and *Berylmys* sp., variances of different variables between groups were tested using an independent sample *t*‐test in SPSS 22.0, and a principal component analysis (PCA) was performed using the log_10_‐transformed craniodental measurements.

## 3 RESULTS

### 3.1 Phylogenetic relationships, divergence time, and species delimitation

**FIGURE 2:**
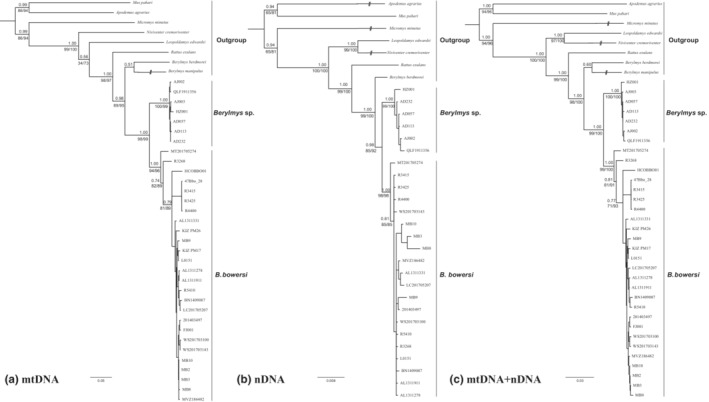
Results of Bayesian phylogenetic analyses of (a) the concatenated mitochondrial‐nuclear genes, (b) the concatenated nuclear genes, and (c) the concatenated mitochondrial‐nuclear genes. Numbers above branches refer to Bayesian posterior probabilities (PP). Numbers below branches refer to SH‐aLRT support values and ultrafast bootstrap values.

We obtained 4950 bp long sequences for each specimen, including 1830 bp of mitochondrial DNA (*Cyt b* [1140 bp] and *COI* [690 bp]) and 3120 bp of nuclear DNA (*RAG1* [1056 bp], *IRBP* [1215 bp] and *GHR* [849 bp]). The ML and BI trees recovered similar topologies, and therefore, only the BI gene trees are shown (Figure 2). In all phylogenetic trees, sequences of *Berylmys* sp. from the Anhui, Zhejiang, eastern China formed a monophyletic clade (SH‐aLRT ≥ 99, Utboot ≥ 99 and PP = 1.00) that was supported as sister to *B. bowersi* from Southeast Asia, and south China (SH‐aLRT ≥ 85, Utboot ≥ 92 and PP ≥ 0.98; Figure 1).

**FIGURE 3:**
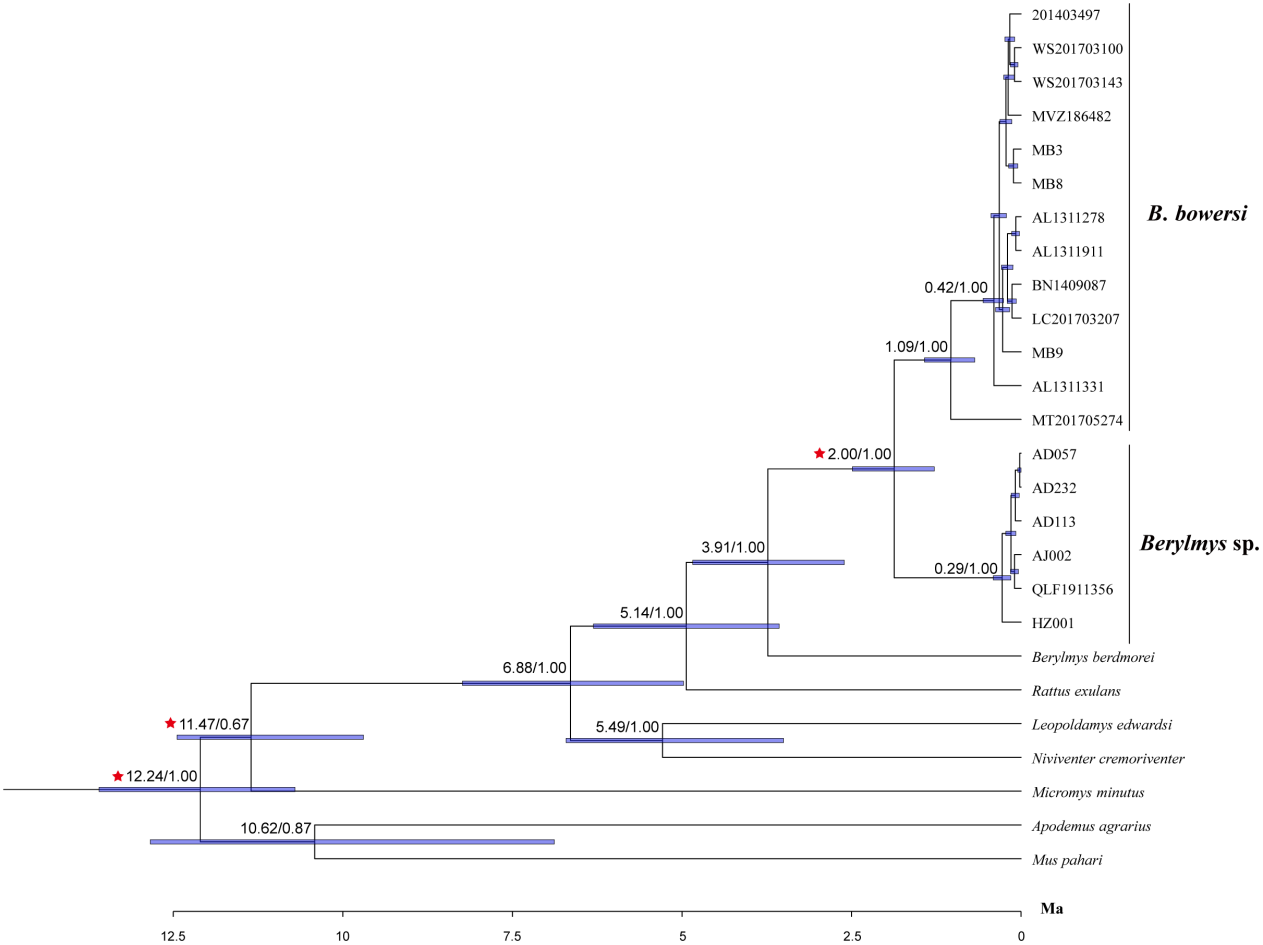
Divergence times estimated using BEAST based on the concatenated dataset of mtDNA and nDNA. Branch lengths represent time. Numbers above the nodes represent the median divergence time and Bayesian posterior probabilities (PP). The three red asterisks indicate fossil‐calibrated nodes.

The BEAST divergence analyses indicated that the ancestral stock of *Berylmys* sp. and *B. bowersi* diverged from *B. berdmorei* at about 3.91 Mya (95% CI = 3.01–4.88 Mya), and the *B. bowersi* and *Berylmys* sp. diverged from each other at approximately 2.00 Mya (95% CI = 1.80–2.36 Mya) (Figure 3). The genetic distance between *Berylmys* sp. and *B. bowersi* on mitochondrial *Cyt b* gene sequence is 8.51% (Table 3). The BPP results consistently supported that *Berylmys* sp. and *B. bowersi* are two distinct species under different algorithms, models, and priors (PP = 1.00) (Table 4).

**TABLE 3:** The uncorrected *p*‐distances in the genus *Berylmys* based on the *Cyt b* gene.

	*Berylmys* sp.	*B. bowersi*	*B. berdmorei*	*B. manipulus*
*Berylmys* sp.				
*B. bowersi*	8.51%			
*B. berdmorei*	10.71%	11.53%		
*B. manipulus*	12.99%	12.82%	11.81%	

**TABLE 4:** Results of BPP analyses for *B. bowersi* and *Berylmys* sp. using different models, algorithms, and priors.

**A10**						
nDNA‐algorithm0	Species delimitation = 1 0 1	Species delimitation = 1 0 5	Species delimitation = 1 0 10	Species delimitation = 1 0 1	Species delimitation = 1 0 5	Species delimitation = 1 0 10
	Heredity = 1 4 4	Heredity = 1 4 4	Heredity = 1 4 4	Locus rate = 1 5	Locus rate = 1 5	Locus rate = 1 5
Guide tree # PP	(*B. bowersi*, *Berylmys* sp.) #1.00	(*B. bowersi*, *Berylmys* sp.) #1.00	(*B. bowersi*, *Berylmys* sp.) #1.00	(*B. bowersi*, *Berylmys* sp.) #1.00	(*B. bowersi*, *Berylmys* sp.) #1.00	(*B. bowersi*, *Berylmys* sp.) #1.00
nDNA‐algorithm1	Species delimitation = 1 1 1 0.5	Species delimitation = 1 1 1.5 1	Species delimitation = 1 1 2 2	Species delimitation = 1 1 1 0.5	Species delimitation = 1 1 1.5 1	Species delimitation = 1 1 2 2
	Heredity = 1 4 4	Heredity = 1 4 4	Heredity = 1 4 4	Locus rate = 1 10.0	Locus rate = 1 10.0	Locus rate = 1 10.0
Guide tree # PP	(*B. bowersi*, *Berylmys* sp.) #1.00	(*B. bowersi*, *Berylmys* sp.) #1.00	(*B. bowersi*, *Berylmys* sp.) #1.00	(*B. bowersi*, *Berylmys* sp.) #1.00	(*B. bowersi*, *Berylmys* sp.) #1.00	(*B. bowersi*, *Berylmys* sp.) #1.00
**A11**						
nDNA‐algorithm0	Species delimitation = 1 0 1	Species delimitation = 1 0 5	Species delimitation = 1 0 10	Species delimitation = 1 0 1	Species delimitation = 1 0 5	Species delimitation = 1 0 10
	Heredity = 1 4 4	Heredity = 1 4 4	Heredity = 1 4 4	Locus rate = 1 5	Locus rate = 1 5	Locus rate = 1 5
Delimited species [PP]	*B. bowersi* [1.00]; *Berylmys* sp. [1.00]	*B. bowersi* [1.00]; *Berylmys* sp. [1.00]	*B. bowersi* [1.00]; *Berylmys* sp. [1.00]	*B. bowersi* [1.00]; *Berylmys* sp. [1.00]	*B. bowersi* [1.00]; *Berylmys* sp. [1.00]	*B. bowersi* [1.00]; *Berylmys* sp. [1.00]
nDNA‐algorithm1	Species delimitation = 1 1 1 0.5	Species delimitation = 1 1 1.5 1	Species delimitation = 1 1 2 2	Species delimitation = 1 1 1 0.5	Species delimitation = 1 1 1.5 1	Species delimitation = 1 1 2 2
	Heredity = 1 4 4	Heredity = 1 4 4	Heredity = 1 4 4	Locus rate = 1 10.0	Locus rate = 1 10.0	Locus rate = 1 10.0
Delimited species [PP]	*B. bowersi* [1.00]; *Berylmys* sp. [1.00]	*B. bowersi* [1.00]; *Berylmys* sp. [1.00]	*B. bowersi* [1.00]; *Berylmys* sp. [1.00]	*B. bowersi* [1.00]; *Berylmys* sp. [1.00]	*B. bowersi* [1.00]; *Berylmys* sp. [1.00]	*B. bowersi* [1.00]; *Berylmys* sp. [1.00]

### 3.2 Morphological analyses

**FIGURE 4:**
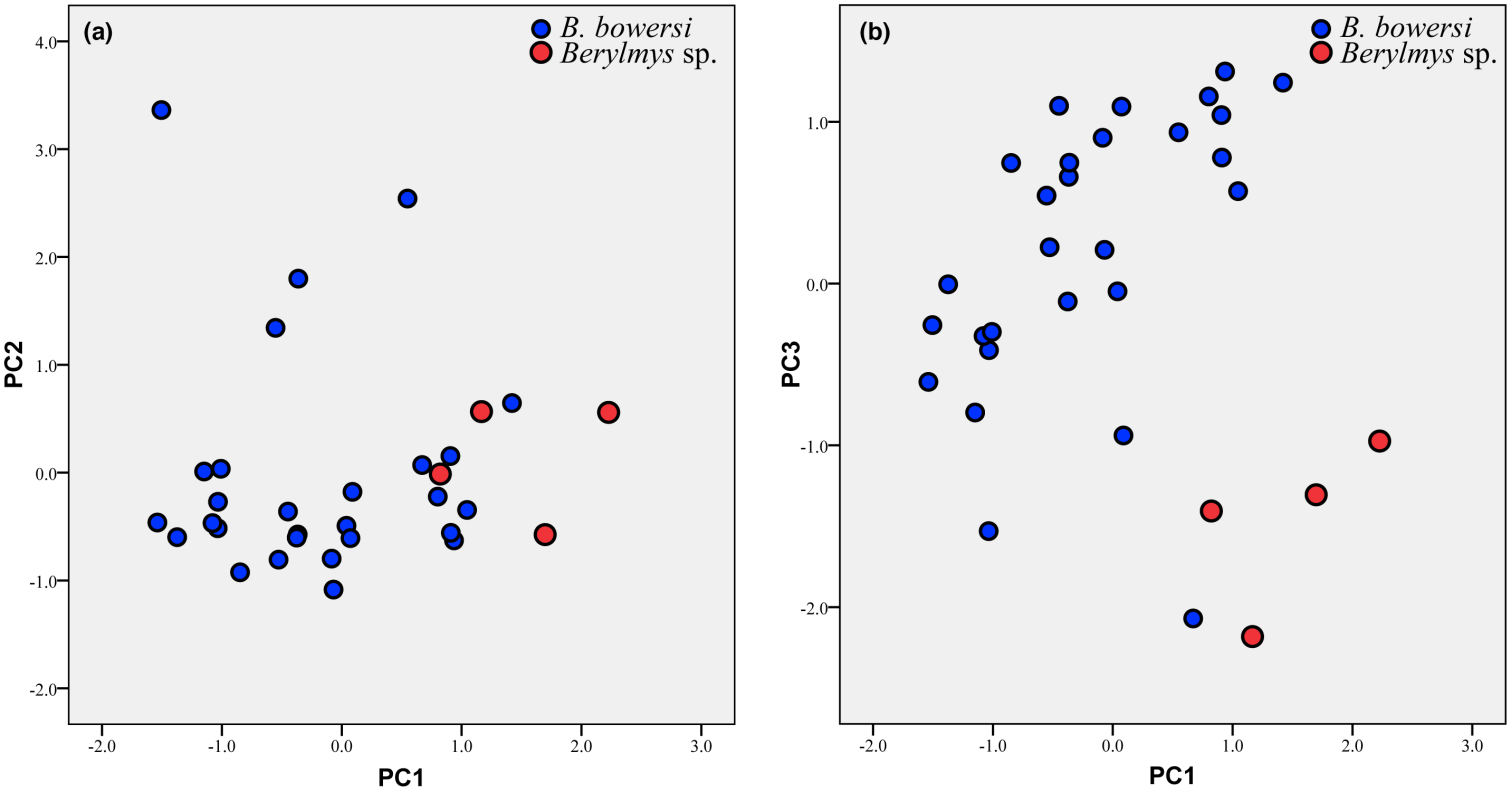
Plot of scores on (a) PC1 and PC2 and (b) PC1 and PC3 from PCA of 23 log10‐transformed craniomandibular variables.

The external and skull measurements are given in Table 5. The mean values of most of the measurements of *Berylmys* sp. are larger than those of *B. bowersi*, and differences between GLS, PPL, NSL, BCH, LAB, ML, and LLTR are significant (*p* < .05). But the *B. bowersi* has relatively larger EL, LIF, IOB, LUM, GBFM, and LLM though partially overlapped. The results of the PCA analysis based on 23 craniodental measurements showed that the first three principal component eigenvalues were greater than 1, accounting for 79.24% of the total variation (Table 6). The first principal component (PC1) was positively correlated with all variables. The second principal component (PC2) was highly positively correlated with LLM, LUM, LPB, and M^2^–M^2^ (loading > 0.750). The third principal component (PC3) was highly positively correlated with RSW and IOB (loading > 0.594) only. A plot of PC1 and PC2 showed *Berylmys* sp. is partially overlapped with *B. bowersi* (Figure 4a), but the former mostly lied on the positive region of PC1, indicating *Berylmys* sp. had a relatively larger skull. In the plot of PC1 and PC3, *Berylmys* sp. was well separated from *B. bowersi* (Figure 4b), and it occupied the positive region of PC1 and negative region of PC3, reflecting its larger skull, wider RSW, and narrower IOB compared with that of *B. bowersi*.

**TABLE 5:** The external and skull measurements (mm) of *B. bowersi* and *Berylmys* sp., including mean values, standard deviations, range, and sample size.

	*B. bowersi*	*Berylmys* sp.
Body weight	265.28 ± 93.09	359.02 ± 75.55
	121.20–510.00; 27	259.11–471.94; 4
Body length	210.30 ± 24.96	223.00 ± 16.69
	160.00–260.00; 28	195.00–250.00; 6
Tail length	235.74 ± 28.80	239.83 ± 17.17
	174.00–301.00; 27	219.00–260.00; 6
Hindfoot length	48.96 ± 3.49	53.55 ± 2.16
	41.00–55.00; 28	49.93–56.57; 6
Length of ear	31.18 ± 3.64	28.98 ± 4.00
	20.00–37.00; 28	22.00–33.27; 6
Greatest length of skull	50.84 ± 4.21	53.67 ± 4.33
	42.10–58.47; 28	46.77–60.59; 6
Palatal length	27.08 ± 1.91	27.41 ± 1.79
	24.46–31.23; 28	24.94–30.47; 6
Postpalatal length	18.82 ± 1.68	19.79 ± 2.05
	15.65–21.80; 28	16.81–23.40; 6
Length of palatal bridge	9.45 ± 1.11	10.27 ± .53
	7.96–12.17; 28	9.72–11.11; 6
Length of incisive foramina	9.30 ± 0.78	8.80 ± 0.76
	8.12–11.14; 28	7.80–9.93; 6
Zygomatic width	24.99 ± 1.25	25.23 ± 1.30
	23.04–27.32; 28	23.28–27.32; 5
Braincase width	21.19 ± 0.76	21.24 ± 0.34
	19.85–22.82; 28	20.59–21.55; 5
Mastoid width	19.61 ± 0.91	19.85 ± 0.56
	17.90–21.69; 28	18.95–20.56; 6
Interorbital breadth	7.87 ± 0.38	7.60 ± 0.17
	7.02–8.80; 28	7.31–7.81; 6
Maximum width across the upper second molars	10.53 ± 0.51	10.76 ± 0.48
	9.63–12.05; 28	10.22–11.65; 6
Least internal breadth between first molars	4.53 ± 0.35	4.78 ± 0.13
	3.87–5.19; 28	4.54–4.92; 6
Rostrum width	7.91 ± 0.70	7.96 ± 0.13
	5.15–8.91; 28	7.81–8.21; 6
Length of upper tooth row	26.80 ± 1.90	27.34 ± 1.62
	24.13–30.99; 28	24.79–29.78; 6
Length of upper molars	9.20 ± 0.90	8.94 ± 0.35
	8.24–11.92; 28	8.29–9.44; 6
Nasal length	19.77 ± 1.82	21.63 ± 2.00
	17.49–23.38; 28	19.42–25.20; 6
Braincase height	16.42 ± 0.85	17.39 ± 0.73
	14.44–17.59; 28	15.91–18.12; 6
Length of auditory bulla	7.87 ± 0.53	8.46 ± 0.50
	6.44–9.09; 28	7.63–9.08; 6
Distance between auditory bulla	4.36 ± 0.80	4.44 ± 0.37
	3.42–6.47; 28	3.86–4.87; 5
Greatest breadth of the foramen magnum	7.77 ± 0.38	7.60 ± 0.37
	7.22–8.95; 28	7.12–8.12; 6
Mandibular length	27.68 ± 2.07	34.18 ± 2.89
	24.39–32.86; 28	30.06–38.81; 5
Length of lower tooth row	22.51 ± 1.69	24.30 ± 1.66
	19.59–26.59; 28	21.82–26.86; 5
Length of lower molars	9.16 ± 0.85	9.06 ± 0.38
	8.32–11.76; 28	8.42–9.61; 6
Height of coronoid process	14.84 ± 1.39	15.28 ± 1.33
	12.17–18.72; 28	13.09–17.52; 6

**TABLE 6:** Factor loadings and percentage of variance explained for principal component analysis.

Variables	PC1	PC2	PC3
Mandibular length	0.869	0.362	−0.106
Postpalatal length	0.862	0.250	0.298
Nasal length	0.854	0.316	0.174
Least internal breadth between first molars	0.854	−0.141	0.180
Zygomatic width	0.815	0.242	0.423
Palatal length	0.815	0.418	0.322
Height of coronoid process	0.800	0.391	0.312
Length of auditory bulla	0.791	−0.232	0.116
Greatest length of skull	0.787	0.507	0.156
Length of upper tooth row	0.777	0.515	0.326
Length of lower tooth row	0.767	0.451	0.040
Mastoid width	0.709	0.327	0.328
Braincase height	0.683	−0.605	−0.127
Length of incisive foramina	0.649	−0.041	0.563
Length of lower molars	0.011	0.917	0.192
Length of upper molars	0.006	0.904	0.246
Length of palatal bridge	0.418	0.844	−0.067
Distance between auditory bulla	0.342	0.750	0.065
Maximum width across the upper second molars	0.506	0.715	0.224
Braincase width	0.525	0.626	0.335
Greatest breadth of the foramen magnum	0.036	0.568	0.407
Rostrum width	0.218	0.145	0.596
Interorbital breadth	0.168	0.416	0.594
Eigenvalue	13.223	3.946	1.086
Variance explained (%)	57.493	17.028	4.722

### 3.3 Morphological diagnosis

Because the distribution range of *Berylmys* sp. includes the type locality of *B. b. latouchei*, one of the junior synonyms of *B. bowersi*, we compared the morphological features of *Berylmys* sp. with *B. b. latouchei*. In the original description, Thomas (1897) presented several morphological characters to distinguish it from *B. bowersi*: (#1) size large; (#2) upper surface gray with two kinds of soft fur; (#3) hands white above, the fingers naked, feet grayish proximally with white on the digits; (#4) tail brown with white tip, covered with thinly haired; (#5) the line of the fronto‐premaxillary and fronto‐nasal sutures runs straight; (#6) the supraorbital rims are more developed and posterior nares wider and more open than *B. bowersi*.

**FIGURE 5:**
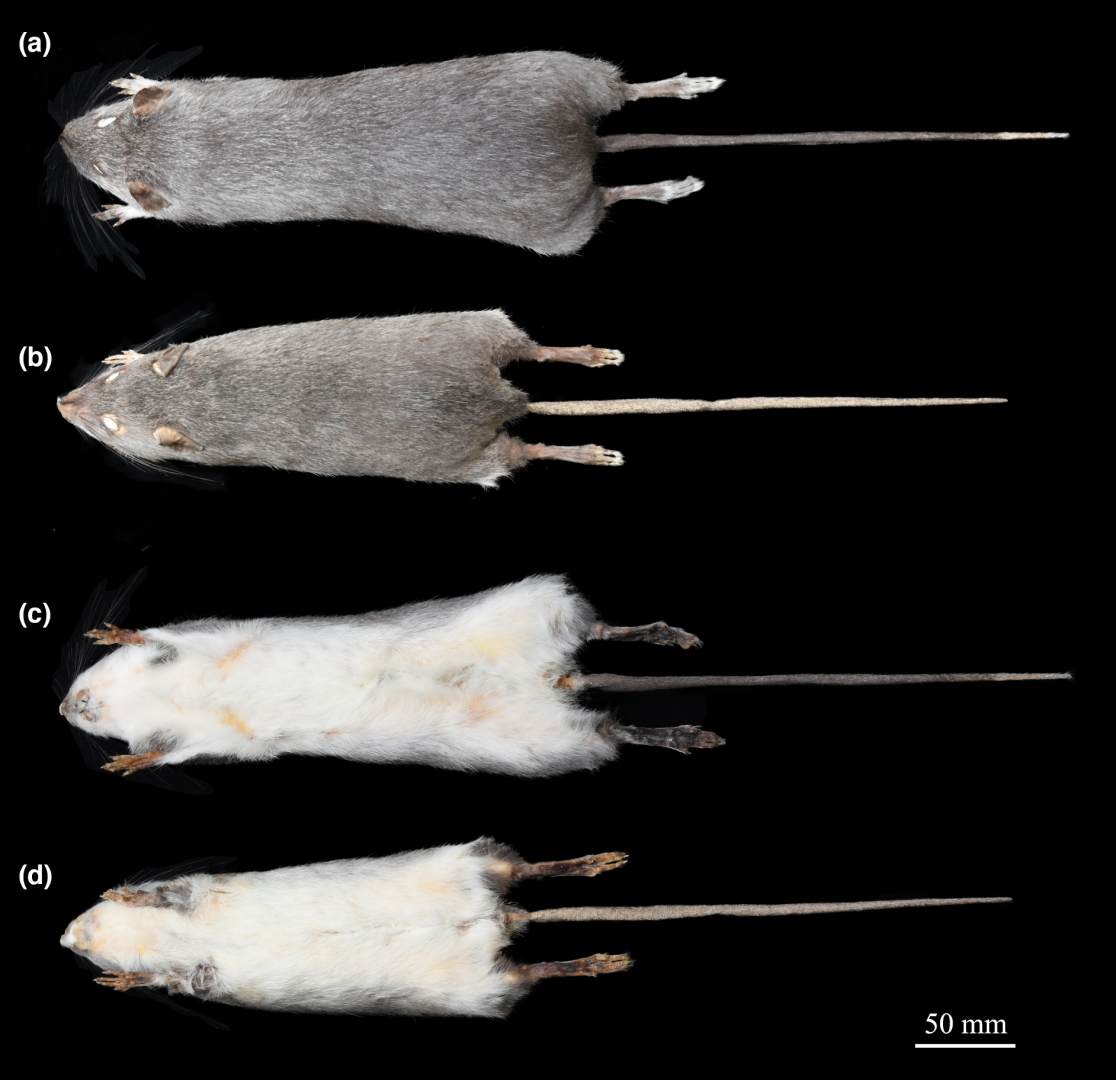
Dorsal and ventral view of *Berylmys* sp. (a and c, AJ002) and *B. bowersi* (b and d, WS201703100).

**FIGURE 6:**
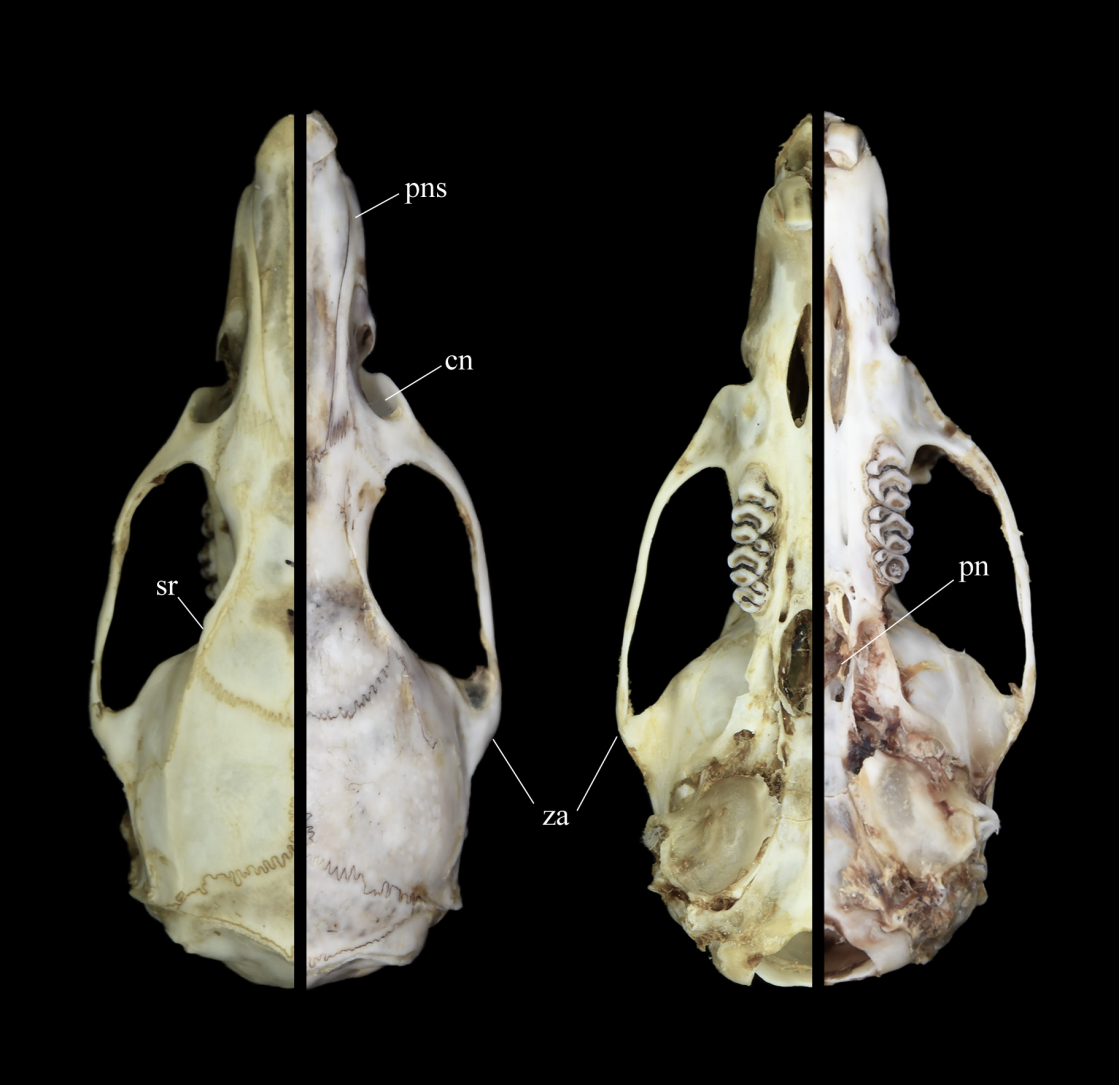
The comparison of dorsal and ventral view of *Berylmys* sp. (left, AJ002) and *B. bowersi* (right, LC201705154), scaled to the same length. The figure legends point to the following characteristics: sutures of premaxillaries and nasals (pns), supraorbital rims (sr), posterior nares (pn), zygomatic notch (cn), and zygomatic arches (za).

Following the original description by Thomas (1897), we examined *Berylmys* sp. and compared it with both *B. latouchei* and *B. bowersi*. The body length of *Berylmys* sp. (HB = 223.00 ± 16.69 mm, range 195.00–250.00 mm) is longer than that of *B. bowersi* (HB = 207.45 ± 26.90 mm, range 147.00–260.00 mm) (#1) (Table 5). The dorsal pelage of *Berylmys* sp. is soft, grizzled gray and consists of two kinds of fur. The first kind is pure white in the bottom half and broadly tipped with dark gray. The second type is pale white in bottom and light gray hair with a white tip, rather slender and intermixed among the first kind. The dorsal fur of *B. bowersi* is coarser and uniformly dark chocolate brown, and appears browner in color (#2) (Figure 5). The hands of *Berylmys* sp. are covered with white fur, and the fingers are almost naked. The digits of the hind feet of *Berylmys* sp. are covered with long white hairs, appearing white; while only short white hairs on the margin of the digits in *B. bowersi*, so the digits of hind feet in the latter appear dusky (Figure 5) (#3). The tail is uniformly brown with white tip, covered with loose white short hairs (#4). The skulls of *Berylmys* sp. (GLS = 54.18 ± 4.57 mm, range 46.77–60.59 mm; BCH = 17.39 ± 0.73 mm, range 15.91–18.12 mm) are larger and higher than that of *B. bowersi* (GLS = 50.45 ± 4.43 mm, range 46.01–54.88 mm; BCH = 16.42 ± 0.85 mm, range 14.44–17.59 mm) (*p* < .05) (#1) (Table 5). The sutures of premaxillaries and nasals of *Berylmys* sp. end evenly instead of being bowed backwards in *B. bowersi* (Figure 6) (#5). The supraorbital rims (= temporal ridges) are more developed, which are continued along the parietals to the outer corners of the intermaxillary. The posterior nares (= pterygoid region) of *Berylmys* sp. are wider and more open than *B. bowersi*, and the zygomatic notch of *Berylmys* sp. is deeper and more distinctive than those of *B. bowersi* (#6). We elaborated that *Berylmys* sp. is distinguished from *B. bowersi* and is consistent with the original description of *B. latouchei* (Thomas, 1897). Thus, *Berylmys* sp., the populations in eastern China, are *B. latouchei*, and it should be a valid species.

**FIGURE 7:**
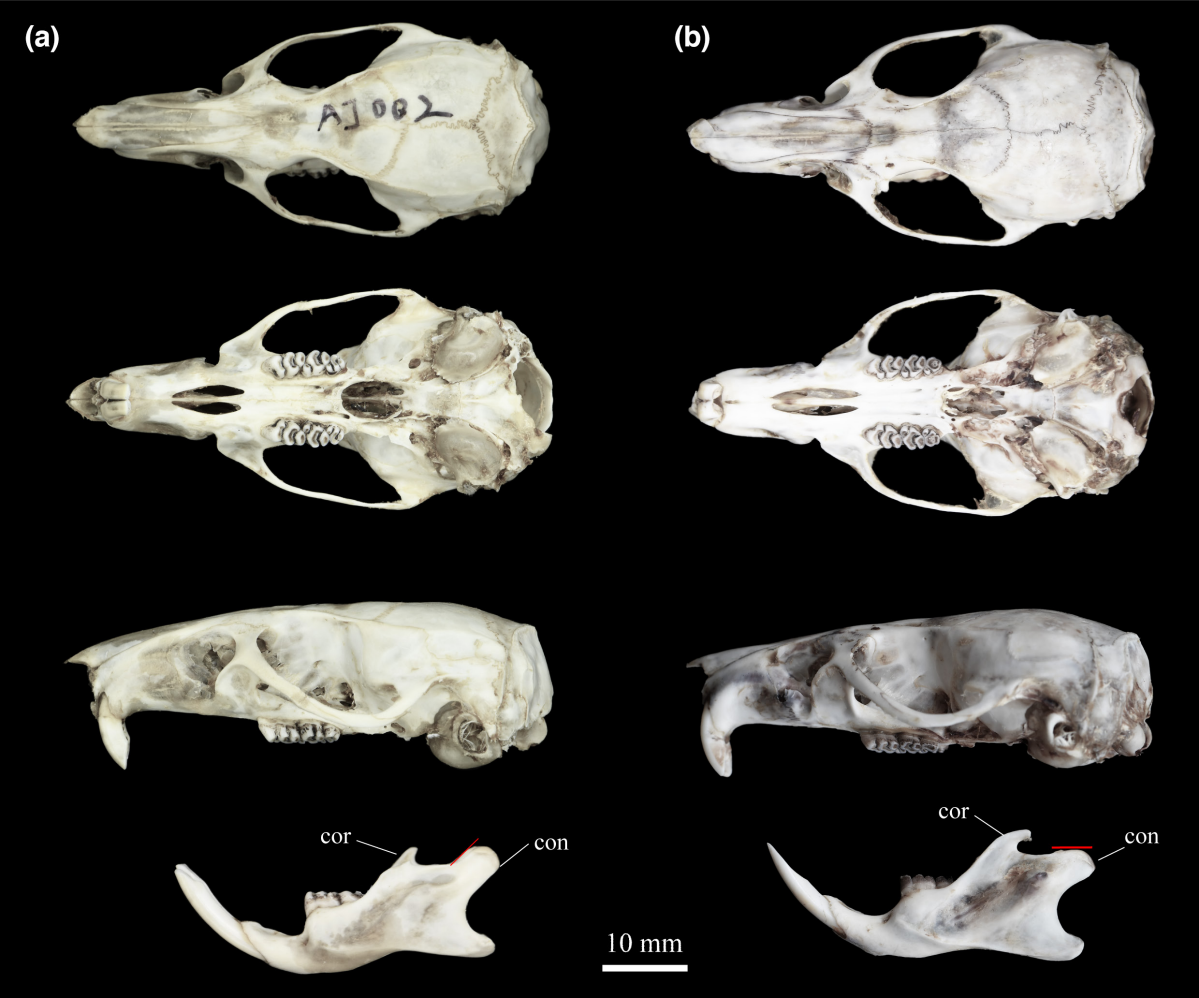
Dorsal, ventral, and lateral views of the skull and lateral views of the mandible of *B. latouchei*. (a, AJ002) and *B. bowersi* (b, LC201705154). The figure legends point to the following characteristics: coronoid process (cor), condyloid process (con), and angle of condyloid process represented by the slope of the red line.

Additionally, several morphological features could assist in identification. Hind feet of *B. latouchei* (HF = 53.55 ± 2.16 mm, range 49.93–56.57 mm) are relatively larger, distinguished from that of *B. bowersi* (HF = 48.96 ± 3.49 mm, range 41.00–55.00 mm) (*p* < .01). Ears of *B. latouchei* are evenly rounded but are smaller than *B. bowersi*, different from the original description. Females of *B. latouchei* have eight mammae: one pectoral pair, one postaxillary pair, and two inguinal pairs, which are same as *B. bowersi* and distinguished from that of *B. manipulus*, *B. berdmorei*, and *B. mackenziei*. The IOB of *B. latouchei* (7.62 ± 0.17 mm, range 7.31–7.81 mm) is narrower than *B. bowersi* (7.83 ± 0.38 mm, range 7.02–8.80 mm), and the zygomatic arches are slightly convergent to the posterior, adding to “the essentially triangular appearance” of the skull (Figure 6). Though the skulls of *B. latouchei* are larger, their molars are shorter and wider than that of *B. bowersi* with partial overlaps. The measurements of LUM (8.94 ± 0.35 mm, range 8.29–9.44 mm) and LLM (9.06 ± 0.38 mm, range 8.42–9.61 mm) are smaller than that of *B. bowersi* (LUM = 9.20 ± 0.90 mm, range 8.24–11.98 mm; LLM = 9.16 ± 0.85 mm, range 8.32–11.76 mm), while the measurements of M^2^–M^2^ and BM^1^ in *B. latouchei* (10.76 ± 0.48 mm, range 10.22–11.65 mm; 4.78 ± 0.13 mm, range 4.54–4.92 mm) are slightly longer than *B. bowersi* (10.53 ± 0.51 mm, range 9.63–12.05 mm; 4.53 ± 0.35 mm, range 3.87–5.19 mm). Also, the auditory bulla of *B. latouchei* are bigger (LAB = 8.46 ± 0.50 mm, range 7.63–9.08 mm) than that of *B. bowersi* (7.87 ± 0.53 mm, range 6.44–9.09 mm) (*p* < .05). Comparing the mandible (Figure 7), its length (ML = 34.18 ± 2.89 mm, range 30.06–38.81 mm) and LUTR (24.30 ± 1.66 mm, range 21.82–26.86 mm) are remarkably longer (*p* < .05). The coronoid process of *B. latouchei* is shorter, and the condyloid process is long and broad, pointing to the posterior at nearly 45 degrees; while the coronoid process of *B. bowersi* is slender, tapering, and curved strongly to the posterior and the condyloid process is parallel with the posterior.

Here, based on the collection and the recently acquired specimens available to us, we provided the following diagnosis for *B. latouchei*: A large‐sized rodent (HB = 223.00 ± 16.69 mm; GLS = 54.18 ± 4.57 mm). External morphology and overall shape of skull similar to *B. bowersi*. The dorsal pelage is soft, grizzled gray and consists of two kinds of fur. Hindfoot is largest in *Berylmys* and covered with long white hairs. Skulls are sharply triangular appearance. The sutures of premaxillaries and nasals end evenly, temporal ridges are developed and the pterygoid region is wide and open. The coronoid process is short, and the condyloid process is long and broad, pointing to the posterior at nearly 45 degrees. Females of *B. latouchei* have eight mammae.

## 4 DISCUSSION

Based on morphology, seven specific names (*bowersi*, *latouchei*, *ferreocanus*, *kennethi*, *lactiiventer*, *wellsi*, and *totipes*) have been assigned to the species under the genus *Berylmys* (Bonhote, 1903; Corbet & Hill, 1992; Musser & Carleton, 2005; Osgood, 1932). Except *latouchei* described from eastern China, all other names were described from Southeast Asia. Thomas (1897) described *Mus latouchei* (= *Berylmys latouchei*) based on the specimens collected from Kuatun (= Guadun), Fujian, China, and pointed out that *latouchei* differs from *B. bowersi* by its larger size, especially its longer hindfeet (Osgood, 1932). But subsequent works always considered it as a junior synonym of *B. bowersi* (Allen, 1940; Denys et al., 2017; Musser & Carleton, 2005; Musser & Newcomb, 1983). In this study, both the morphological and molecular analyses indicated *B. latouchei* is distinct from *B. bowersi*. Based on the diagnosis and monophyly phylogenetic species concept (Gutierrez & Garbino, 2018; Mayden, 1997), we recognized *B. latouchei* as a valid species. The species is currently known from north Fujian, south Zhejiang, and southwest Anhui, China, with the Dabie Mountains in west Anhui being the northernmost distribution boundary. Specimens from Sanming, Fujian, and Guangdong were identified as *B. bowersi*, and thus, the Minjiang River likely acted as a genetic barrier between *B. latouchei* and *B. bowersi* (Figure 1).

Our molecular analyses found several considerable divergences in *B. bowersi*. One specimen (MT201705274) from Motuo, Tibet, China remained distinct on the basal position of *B. bowersi*; the genetic distance of *Cyt b* between the specimen and specimens from Yunnan, China (type locality of *B. bowersi*) is 5.24%. Our study also included a specimen (R3268; Pages et al., 2010) from the type locality of *B. ferreocanus* (Trang, Thailand), it also formed a separate clade (Latinne et al., 2013); and the *Cyt b* genetic distance between this specimen and specimens from Yunnan is 5.12%. These results suggest the distinct intraspecific diversification of *B. bowersi*, hence, further studies on the taxonomy of *B. bowersi* are needed.

Most fossils of *Berylmys* were reported from southwest China (*B. bowersi*; Zheng, 1993) and Thailand (*B. berdmorei*; Chaimanee, 1998). Considering the fossil records and current distributions, *Berylmys* possibly originated in western China or Indochinese Peninsula, while the populations in eastern China are more likely the migrators. The divergence time of the ancestral stock of *B. bowersi* and *B. latouchei* with the *B. berdmorei* is estimated for the Middle Pliocene, which corresponds to the rapid diversification of most of the Rattini tribe of muroid rodents (Latinne et al., 2013). The rapid uplift of the Hengduan Mountains and ensuing climate warming (Chen, 1992; Xing & Ree, 2017) in the Middle Pliocene may have led to the divergence of *B. bowersi* + *B. latouchei* and *B. berdmorei*. Meanwhile, it may have caused the ancestor of *B. bowersi* + *B. latouchei* to migrate to eastern China. There are multiple mountains and hills with different layouts in subtropical China that provide topographic heterogeneity and complex habitats (Shen et al., 2019), which act as dispersal corridors to facilitate the dispersal of terrestrial small mammals (Chen et al., 2022). Thus, the ancestor of *B. bowersi* and *B. latouchei* possibly migrated via the southeastern hills to eastern China, a potential migration route supported by several studies (Aplin et al., 2011; Ge et al., 2021).

The split between *B. latouchei* and *B. bowersi* likely occurred in the early Pleistocene (ca. 2.00 Mya), which could attribute to the combined effect of climate change and geological events in the early Pleistocene. The global cooling and desiccating events in the early Pleistocene have been well‐documented (Fujiki & Ozawa, 2008; Webb & Bartlein, 1992). The variable topography in Wuyi Mountains and mountains in southern Anhui and Zhejiang may have provided refuges for them, and led to the divergence of *B. latouchei* and *B. bowersi*. The deep rivers between mountain ridges have been demonstrated as barriers to the dispersal of small mammals (Chen et al., 2022; He et al., 2019). The Minjiang River likely acted as a barrier to the dispersal, resulting in the current distribution pattern of *Berylmys*.

Eastern China, containing many large mountain ranges such as Wuyi Mountains, Tianmu Mountains, and Dabie Mountains, are considered hotspots for speciation and have a high degree of endemism (Lv et al., 2018). In recent years, new small mammal species such as *Crocidua dongyangjiangensis* Liu et al., 2020, *Typhlomys huangshanensis* Hu et al., 2021 and *Chodsigoa dabieshanensis* Chen et al., 2022 have been discovered in the region. Our results together with these studies indicate that small mammal species diversity is considerably underestimated in eastern China. More intensive surveys and systematic revisions are needed to improve our understanding of the biodiversity of the region.

## AUTHOR CONTRIBUTIONS

**Yifan Xu:** Data curation (lead); formal analysis (lead); software (lead); writing – original draft (lead). **Jiangxiao Hu:** Resources (supporting); software (supporting). **Zifan Shi:** Resources (supporting); software (supporting). **Wenwen Chen:** Resources (supporting). **Jiajun Zhou:** Resources (supporting). **Baowei Zhang:** Resources (supporting). **Yong Fan:** Resources (supporting). **Laxman Khanal:** Writing – review and editing (equal). **Xuelong Jiang:** Conceptualization (equal); methodology (equal); writing – review and editing (equal). **Zhongzheng Chen:** Conceptualization (equal); methodology (equal); writing – original draft (supporting); writing – review and editing (equal).

## FUNDING INFORMATION

This research was funded by the Second Tibetan Plateau Scientific Expedition and Research Program (No. 2019QZKK0501), the National Natural Science Foundation of China (No. 31900318), the Project of Biological Resource Survey in Wuyishan National Park, and the University Synergy Innovation Program of Anhui province (GXXT‐2020‐075).

## CONFLICT OF INTEREST STATEMENT

The authors declare there are no competing interests.

## Data Availability

All the New DNA sequences (accession numbers OQ160975–OQ161004, OQ161020–OQ161049, OQ168972–OQ168387) used in this study were deposited in GenBank (https://www.ncbi.nlm.nih.gov/).
